# The crossroads of GIS and health information: a workshop on developing a research agenda to improve cancer control

**DOI:** 10.1186/1476-072X-5-51

**Published:** 2006-11-21

**Authors:** Linda Williams Pickle, Martha Szczur, Denise Riedel Lewis, David G Stinchcomb

**Affiliations:** 1 Surveillance Research Program, Division of Cancer Control and Population Sciences, National Cancer Institute, Bethesda, MD USA; 2 Division of Specialized Information Services, National Library of Medicine, Bethesda, MD USA

## Abstract

Cancer control researchers seek to reduce the burden of cancer by studying interventions, their impact in defined populations, and the means by which they can be better used. The first step in cancer control is identifying where the cancer burden is elevated, which suggests locations where interventions are needed. Geographic information systems (GIS) and other spatial analytic methods provide such a solution and thus can play a major role in cancer control. This report presents findings from a workshop held June 16–17, 2005, to bring together experts and stakeholders to address current issues in GIScience and cancer control. A broad range of areas of expertise and interest was represented, including epidemiology, geography, statistics, environmental health, social science, cancer control, cancer registry operations, and cancer advocacy. The goals of this workshop were to build consensus on important policy and research questions, identify roadblocks to future progress in this field, and provide recommendations to overcome these roadblocks.

## Background

Cancer control researchers seek to reduce the burden of cancer by studying interventions, their impact in defined populations, and the means by which they can be better used [1,2]. This is a multidisciplinary field, including epidemiologists, demographers, statisticians, behaviorists, risk communication experts, and other social scientists. The first step in cancer control is identifying where the cancer burden is elevated, which suggests locations where interventions are needed. It has long been recognized that cancer rates vary by region [3,4], but only recently has it become apparent that local neighborhoods also can have an influence on cancer outcomes (see review by Diez Roux[5]), perhaps through shared environmental exposures and cultural and behavioral factors. The multilevel and multifactorial features of this more complex view of cancer control are best addressed by techniques more complex than univariate maps and simple statistics.

Geographic information systems (GIS) and other spatial analytic methods provide such a solution and thus can play a major role in cancer control. Geographic information science (GIScience), the science behind GIS, is multidisciplinary, encompassing topics in geography, cartography, statistics, computer science, and subject-specific fields. The application of GIScience to the health field is fairly new but growing rapidly.

Not only are the research communities in cancer control and GIScience diverse and multidisciplinary, but so are those who use or put into practice the findings of the researchers. For example, state epidemiologists combine information on the geographic distribution of cancer rates and knowledge of local sociodemographic patterns with research on the most effective communication methods to design a program to increase the use of cancer screening in their area. Similarly, members of a local community will sometimes raise a warning about apparently high cancer rates in their area, which will lead to systematic enumeration of cancer cases, etiologic studies and subsequent interventions by appropriate health agencies. All of these diverse users of geographic and statistical cancer information have a stake in the quality of GIScience and cancer control research, for poor quality data or incorrect methods will lead to misguided expenditure of scarce resources and will not reduce the cancer burden as hoped.

Conferences to date have focused on cancer control or GIScience, or on GIScience and health in general, but few have included experts in both areas. Similarly, few workshops have included stakeholders from the research, data provider, and user communities. Bringing representatives of all of these communities together has obvious advantages, but ensuring that those who have different points of view, areas of expertise, and sets of jargon, can communicate effectively also presents challenges.

We report here on a workshop held June 16–17, 2005, to bring together experts and stakeholders to address current issues in GIScience and cancer control. A broad range of areas of expertise and interest was represented, including epidemiology, geography, statistics, environmental health, social science, cancer control, cancer registry operations, and cancer advocacy. The goals of this workshop were to build consensus on important policy and research questions, identify roadblocks to future progress in this field, and provide recommendations to overcome these roadblocks.

The workshop was jointly organized by the Division of Cancer Control and Population Sciences (DCCPS) of the National Cancer Institute (NCI) and the National Library of Medicine (NLM), both part of the National Institutes of Health (NIH). NCI has a longstanding interest in the geographic patterns of cancer [4] and a growing program in GIS (see gis.cancer.gov), and NLM has supported the GIScience community by including journals of interest in its searchable bibliographic databases and by developing several tools to explore geospatial data (see toxmap.nlm.nih.gov).

### Preparation for the workshop

Our first step in planning the topics and structure for the workshop was to conduct a series of telephone interviews in 2004 and early 2005 with experts in areas relevant to GIScience. Their opinions led us to structure the workshop around three main focus areas: (1) data issues, such as the need for particular types of data and the tension between protecting privacy and obtaining potentially-identifiable health data for GIS analysis; (2) computer or information resources that can be shared among interested parties; and (3) collaboration across agencies or between federal, state and local partners.

We also used the results of two focus groups conducted in 2004 by Dr. Thomas Richards, a Medical Officer from the Division of Cancer Prevention and Control, Centers for Disease Control and Prevention (CDC). Participants in the focus group – state-based cancer control practitioners and partners, geographers, and spatial statisticians – were asked to help define a future GIS research agenda by identifying GIScience priorities. Dr. Richards was invited to present his conclusions at our workshop so that the participants would benefit from the earlier discussions by GIS experts.

Participants in the workshop were invited from the federal, state, cancer registry, academic and cancer advocate communities. Of the 85 participants, 50% were from the federal government, representing the Department of Health and Human Services, the Department of Agriculture, the Environmental Protection Agency, Bureau of the Census and the U.S. Geologic Survey. Most of the 25 academic researchers in attendance were NCI grantees. Fourteen participants were from cancer registries, representing nearly all of the registries in the NCI Surveillance, Epidemiology and End Results (SEER) program. Three members of cancer advocacy groups also attended. Areas of expertise of these attendees included GIS, geography, cartography, epidemiology, cancer control, social science, demography, statistics, computer science and environmental science.

### Format of the workshop

During the first day, experts presented their views of the state of the science in GIS and cancer control. Additional presentations by NIH staff provided background on GIS and cancer control and updates on activities at NCI and NLM in this area. Participants then met in groups and identified challenges in each of the three focus areas. On the second day, participants voted to set priorities among these challenges and met in small groups to brainstorm solutions. Participants self-selected the single topic they wished to discuss. In an effort to have a balance between government, academic and other members, some realignment of the small groups was necessary, resulting in small groups of 5–15 participants each. In the last session of the workshop, a representative of each group presented a summary of their recommendations to everyone in a plenary session. Final recommendations presented here are the results of the discussions within the small groups, with input from participants in this last plenary session.

### Presentations to set the stage

#### Background

We begin by summarizing the presentations that framed the context and objectives of the workshop. Mr. William Davenhall, the Health and Human Services Solution Manager from ESRI, a key force in GIS software development, began by defining GIS and illustrating how it can be used in various biomedical and health areas. Dr. Ben Hankey, Cancer Statistics Branch Chief of the Surveillance Research Program (SRP) at the National Cancer Institute at the time of the workshop, defined cancer control and focused on GIS's use in this area. Dr. Thomas Richards of the Centers for Disease Control and Prevention (CDC) then elaborated on how GIS can benefit cancer control and articulated some priorities in GIScience identified by expert focus groups convened at CDC. Other presentations updated the audience on GIS activities at NCI and NLM and provided an overview and examples for each of the main areas for discussion – data issues, shared resources, and collaboration.

In the keynote address, Mr. Davenhall described the state of the art in GIS and how its emerging use in the biomedical and health arena is just beginning to catch up with its use in business domains, such as finance and mining. He introduced GIS as way to communicate, collaborate, and connect – both across data and people – with the goal of building common understanding. He showed examples of how GIS technology can be used to integrate administrative health data, health facility data, and clinical data. Health-related GIS programs are growing in graduate schools and hospitals with labs and programs in geoinformatics, health geographics, remote sensing, and geospatial medicine. Although use of GIS in biomedical research has great potential, it is still embryonic in its application. Challenges include overcoming the belief that "geography does not matter" and that "adding geography is labor intensive and costly." Mr. Davenhall offered some ideas on how to overcome these barriers, such as adding geographers to research teams, promoting the collection and storage of accurate and complete address information as well as geocoding to a specific location, integrating more "lifestyle" and socio-demographic data, developing new spatial referencing systems for human anatomy, encouraging greater use of GIS in clinical trials, using GIS to link large medical data sets, and creating Centers of Excellence in Geospatial Cancer Research. The presentation's concluding message was a quote from NCI's Director at the time, Dr. Andrew von Eschenbach, which encouraged us to "...focus on enabling technologies and gain strength from all sectors."

In the second presentation, Dr. Ben Hankey made the case that cancer control provides myriad opportunities to use GIS methods. He defined cancer control as "the reduction of cancer incidence, morbidity and mortality through an orderly sequence from research on interventions and their impact in defined populations to the broad systematic application of the research results" and cancer control research as "the conduct of basic and applied research in the behavioral, social, and population sciences that, independently or in combination with biomedical approaches," accomplishes this goal. He also explained that cancer surveillance was the aspect of cancer control that measures cancer incidence, mortality, and morbidity along with "patient survival, risk factors, health system and lifestyle factors, screening utilization, genetic predisposition and environmental exposures by demographic factors and geographic area." He then discussed ways to expand the scope of cancer surveillance research by collecting data on many of these cancer-related factors on cohorts of cancer patients, such as those collected by the NCI-sponsored cancer registries in the Surveillance, Epidemiology and End Results (SEER) Program. Current GIS areas of study in SRP include ecologic surveillance (i.e., patterns of rates reflecting differential impact of cancer control interventions based on social economic status measures), identification of health disparities and problem areas, geographic focus of cancer control efforts (e.g., disseminating cancer information by state or county), modeling/predicting cancer rates using ecologic data for a subset of the population, cancer cluster identification, assessment of medical facility placement, and ecologic correlations (i.e., impact of cancer control interventions.) He identified some of the key challenges in implementing GIS methods as restricted data access due to patient confidentiality, obtaining quality geocoded data at a local level, and obtaining ecologic data.

Dr. Thomas Richards then summarized the findings and recommendations from the 2004 CDC focus groups. The primary question addressed in the focus groups was "How can comprehensive cancer control benefit from an enhanced focus on GIScience?" Although the focus group results quickly identified a communication problem – cancer control staff and GIS users "speak different languages" – they did identify how maps could contribute to cancer control when used as a part of descriptive epidemiology, in newsletters/annual reports, to tell a compelling story, and for "quick facts" communications with legislators/media. Dr. Richards referenced a chart developed by Dr. Myles Cockburn from the Department of Preventive Medicine at University of Southern California, which presented examples of the current successes and future opportunities for GIS application in each of the major cancer control activities (Mobilizing Support, Assessing/Addressing Cancer Burden and Utilizing Data/Research). For example, for the task of information dissemination as it relates to "Mobilizing Support," there have been successes in publishing cancer maps and development of web-based cancer mapping tools. Challenges to be addressed include how to present data in a way that meets the information needs of the user while clearly and accurately portraying the underlying data.

#### Current GIS activities within NCI and NLM

Dr. Linda Pickle, Senior Mathematical Statistician in the Division of Cancer Control and Population Sciences of NCI, summarized geospatial activities at NCI. The National Cancer Institute has a long-standing interest in the geographic patterns of cancer [4] and a growing program in GIS (see gis.cancer.gov). Researchers at NCI are actively engaged in projects in the areas of GIS database development, spatial data analysis, and geovisualization tools development, particularly for communication of georeferenced cancer statistics.

An example of an NCI GIS project is the Long Island GIS, one of a series of initiatives within the Long Island Breast Cancer Study Project. This Congressionally-mandated effort is designed to understand high breast cancer incidence rates on Long Island, NY. The Long Island GIS was designed to study the potential relationships between environmental exposures and breast cancer, but the system can be used to study other diseases as well. The Long Island GIS includes over 80 datasets and a full suite of analytic software. ESRI's ArcGIS software is supplemented by extensions, including tools to calculate age-adjusted rates, interpolate the number of events for specified areas, smooth the mapped rates by either an empirical Bayes or nonparametric method, identify clusters of similar rates, mask sparse data and link to statistical software. The web site [6] currently presents publicly-available information and will soon include an interactive map capability. Confidential data and licensed software are accessible by approved researchers through a secure computer system.

Dr. Pickle also described NCI's wide-ranging research in the area of spatial data analysis. For example, methods have been developed to estimate the potential for specific pesticide exposure in agricultural areas by translating satellite images of crop lands to land cover maps, then estimating the probable type and dose of pesticide for each crop [7]. This is an example of how a GIS can provide exposure estimates that are not available from any other source. Now that images of the surface of the U.S. are available from LandSat satellites for the past 30 years, it should be possible to estimate historic pesticide exposures using these methods.

Hierarchical statistical models also have been developed to predict cancer incidence for all U.S. counties using data from a limited number of high-quality cancer registries [8]. These spatial prediction models are being extended to project case counts from the latest year of available data to the next calendar year for planning purposes. Cluster identification methods first developed at NCI [9] have been extended to identify elliptical as well as circular clusters and are being extended for application to survival rates and ordinal data[10-12].

A number of these developments have been used to improve the communication of georeferenced statistics and dissemination of cancer data to the cancer control community and to the public [13,14]. For example, the NCI program in geovisualization builds upon cognitive research at the National Center for Health Statistics to study effective map design [15]. Collaboration with academic researchers has led to the development of tools for mapping, exploratory spatial data analysis and enhancements to GIS software [16-20].

Ms. Marti Szczur, Deputy Associate Director of the Specialized Information Services Division at National Library of Medicine (NLM), gave an overview of the NLM resources of particular interest to cancer control researchers. Although NLM is best known to researchers for its MEDLINE collection of over 15 million bibliographic citations [21], Ms. Szczur introduced several other web-based information resources, including MedlinePlus [22], which has more than 700 health topics pages that link to relevant information from NIH and other authoritative sources; Genetics Home Reference [23], which provides information about genetic conditions and the genes or chromosomes responsible for those conditions; and ClinicalTrials.gov, which contains information about federally- and privately-supported clinical research in human volunteers. She also summarized the resources available in the NLM Toxicology and Environmental Health Program, which includes TOXNET [24], a collection of databases with peer-reviewed information about hazardous substances; Household Products Database [24] with information on the potential health effects of over 6,000 common commercial products; an occupational health database about the health effects of exposure to chemicals at work; and a GIS application, TOXMAP [24], which integrates maps of toxic release sites with access to related chemical and bibliographic databases. While MEDLINE records are not geo-coded, TOXMAP extracts the text names of geographic features (e.g., cities, towns, counties, rivers) displayed in the map window and searches the MEDLINE record titles and abstracts looking for matches. This provides a starting point for reviewing research citations related to the chemical and geographic area of interest. She invited the participants to think about what future library services would help them in their research quests (e.g., specialized/pre-formulated MEDLINE searches, GIS tools inventory/locator, additional GIS-related journals in PubMed, extended functionality and/or data in TOXMAP.)

### Topic-specific presentations

To stimulate targeted discussion from participants and provoke creative thinking, invited speakers in each of the three focus areas presented their views of key issues related to data, resources, and collaboration.

#### Data issues

Dr. Virginia Lee, a medical officer and Team Leader of the GIS program at the Agency for Toxic Substances and Disease Registry (ATSDR)/CDC, discussed the strengths and challenges of linking multiple data sources for decision making. She identified use of GIS for public health decisions, such as determining geographic distribution of diseases, analyzing spatial/temporal trends, mapping populations at risk, stratifying risk factors, planning/targeting interventions, and monitoring diseases and/or interventions over time. She gave an overview and examples of the type of data integrated into a GIS for use in public health decision making, such as Census base map data, Census population data, health related datasets, resources and environmental data sets. An example of the challenges associated with these data is an analysis of cancer patterns over time by ZIP code, because boundaries of these units frequently change and are approximated geographic boundaries.

Dr. Gerard Rushton, professor of geography and public health from University of Iowa discussed confidentiality restrictions and methods to allow analysis and presentation of potentially-identifiable health data. The Health and Human Services (HHS) Healthy People 2010 report [25] identifies "a major challenge in the coming decade will be to increase public access to GIS information without compromising confidentiality." Dr. Rushton addressed two approaches to privacy protection:

• The application of approved disclosure limitation methods before releasing the data to the public (e.g., spatial and environmental masks that hide the exact location of mapped data points); and

• Control of the computing environment with an "agent-based" approach that allows the restricted data to be processed by intelligent software systems (agents) on the server side (behind a secure firewall) rather than on the client side.

#### Shared resources

Dr. Geoffrey Jacquez, from BioMedWare, Inc., discussed how the integration of new technologies (e.g., sensors, software, location-based devices, distributed data) with new ways of thinking (e.g., micro medical geography) can lead to rapid advances in the population sciences needed to achieve the NCI goal of "eliminating suffering and death due to cancer by 2015." One of the key challenges Dr. Jacquez raised is how to account in geographic studies for problems such as latency (i.e., the time lag between exposure and diagnosis, which can be more than 20 years), human mobility (i.e., the average American moves every 5 to 7 years), and variability in individuals' exposures. Space-time analysis software that links distributed data sources, customizable software platforms that support visualization, exploratory data analysis (EDA), and modeling are tools that can be applied to these challenges.

Dr. Nina Lam, the Richard J. Russell Professor of Geography at Louisiana State University and President of University Consortium for GIScience (UCGIS), discussed problems in environment health research, which include the uncertainties associated with existing methods and data. Sources of uncertainties include errors in collecting and recording health and demographic data, variations in the choice and method of applying analytic tools such as general and focused cluster detection, and problems in interpreting results. Her proposed solution was a spatial analytic framework with five integrated, interactive modules: visualization and measurement, cluster detection, focused exposure modeling, scale sensitivity analysis, and decision support.

#### Collaboration

Mr. Charles Reynolds, Special Expert at the HHS Substance Abuse and Mental Health Services Administration (SAMHSA), described the agency's web-based system that allows the user to analyze federal and local resources down to the neighborhood level. SAMHSA works collaboratively with states, national and local community-based and faith-based organizations, public/private sector data providers, and other funding agencies on this system, which enables their project officers to determine whether grants are being awarded to the communities where they are most needed and helps grantees reach targeted populations. Mr. Reynolds demonstrated how this GIS tool allows users to integrate their internal data and create custom reports. It demonstrates an innovative use of GIS that may be applicable within the cancer control community.

Dr. Eugene Lengerich, Associate Professor, Division of Epidemiology at The Pennsylvania State University's (PSU) College of Medicine and Director of Community Education and Outreach at the Penn State Cancer Institute continued the discussion of collaboration by identifying assumptions for successful collaborations in public health. These assumptions are:

• A shared mission exists;

• Each entity makes a unique, necessary contribution;

• Synergism is present;

• A common language is spoken; and

• It is possible to draw upon additional resources (e.g., financial and/or social capital).

Dr. Lengerich described a successful community-based participatory research collaboration at the Northern Appalachia Cancer Network, a regional member of the Appalachia Community Cancer Network, in which the application of GIScience was an effective component in evidence-based community interventions. He also discussed how diffusion of a model GIS/Atlas into state cancer control activities can help researchers investigate individual and contextual factors associated with geographic distribution of cancer incidence at the state level. This is particularly true for Comprehensive Cancer Control efforts, where partnerships between public and private sector shareholders are established and activities coordinated in order to make the most effective use of limited resources to promote cancer prevention, improve cancer detection, increase access to health and social services, and reduce the burden of cancer.

### Specific issues and recommendations

Following these presentations, workshop participants identified issues for cancer control and GIS and voted on the topics of greatest importance (Table 1). Several issues were so similar that they were combined to form six major topics chosen for discussion on the second day. In addition, two topics (Table 1, #7 and #8) were added to the discussion list by special request of several participants, even though they had not received higher number of votes than some other topics. We summarize below the issues chosen by the participants and their recommended solutions, presented in the order of the number of votes received, with the number of votes shown in parentheses.

**Table 1:** Issues identified by workshop participants as important for GIS and cancer control, with votes for each.

	**Issue**	**Total Votes**
	***Major Issues Discussed***	

1	Develop methods to ensure privacy and confidentiality while allowing access, especially with small data sets. Encourage collaborations among agencies, ethicists, HIPAA specialists, "maskers" to reduce ethical barriers to sharing data.	54
2	Develop tools and theory to deal with time and spatial temporal aspects. How do we get geo dynamic data (e.g., spatial/temporal, residential history)?	43
3	Create a searchable and user-friendly one-stop portal for data, boundaries, references, and tools. Foster collaboration among data holders and GIS developers to create metadata that are standardized, understandable, and usable by multiple stakeholders.	31
4	Build a critical mass of multi-disciplinary scientists to work together in a Center of Excellence dedicated to developing theoretical and practical GIS studies	19
5	Develop strategies and tools for handling and communicating uncertainty.	18
6	Create methods to use census data more effectively in combination with health data collected.	15
7	Determine that community-based participatory research is the approach that should be used for GIS when used as a tool for cancer control.	14
8	Fund and support "high-risk, high-gain" tool and resource development.	9

	***Additional Issues Raised***	

9	Standardize and develop best practices for statistics (e.g., cluster analyses); eliminate overlap with regard to display mechanisms, technologies, and methods.	14
10	Encourage collaboration among cancer control specialists, GIS experts and policy makers to enable them to understand GIS information (e.g., evaluate information they receive that is conflicting or contradictory, develop policies that will be likely to control cancer, enable stakeholders to communicate that information and those policies, and to give them GIS information that applies to their geopolitical boundaries).	14
11	Support the development of theory-based methodologies that quantify or assess the total uncertainty in a GIS cancer analysis.	13
12	Improve the accuracy of geocoding in the short- and long-term.	13
13	Develop standards and methods to ensure data quality to avoid erroneous inferences from complex data.	12
14	Focus broadly on goal of reducing cancer burden using GIS methods (e.g., by facilitating how current exploratory tools can be used in interventions and clinical trials).	12
15	Train both information users and providers in effective ways to transfer knowledge.	11
16	Develop tools, such as simple flexible mapping programs, for users at all levels.	10
17	Foster collaboration between GIS developers and the public to enable the public to understand GIS data, use GIS data, become informed about GIS findings and health and exposure and health access patterns in their geographical areas; identify public health issues of concern to community; develop GIS systems responsive to those needs.	10
18	Encourage collaborations among multi-disciplinary groups that develop and disseminate rigorous spatial statistical methods.	10
19	Support collaboration among GIS academics and other GIS experts and public health, academic, and agencies that do not have the expertise to develop mentorship and training programs for those that do not have them, cannot afford them, or are too small to have GIS experts.	9
20	Improve information on data quality (e.g., robustness of data feeding into software; Census data and affect on rates and data compatibility and sources).	8
21	Foster collaboration among agencies and between data holders and GIS developers to facilitate and standardize sharing data that can be used in GIS systems.	8
22	Develop data integration strategies (e.g., integrate data from multiple stakeholders).	7
23	Establish methods to deal with practical problems (e.g., political issues, economic effects, need to define boundaries such as school districts.	6
24	Develop web-based, feasible, and usable tools for handling and managing GIS data.	5
25	Develop interdisciplinary tools that enable collaboration and ability to work effectively with geospatial information.	5
26	Facilitate collaborations among GIS developers, program evaluators, and funders to evaluate whether money invested in GIS and other health programs leads to improvements in cancer control.	5
27	Support collaborations between GIS developers and health professionals that lead to improved understanding of health patterns in communities.	5
28	Create interdisciplinary collaborations to improve ability to conduct small area studies.	4
29	Encourage collaboration between the U.S. Postal Service and other agencies to make postal code information accessible, usable, and useful.	4
30	Develop simplified standards-based, automated data merging tools.	3
31	Develop non-spatial resource for GIS, including imagery library.	3
32	Encourage 3D visualization and knowledge spatio-temporal pattern analysis.	2
33	Develop communication and education strategies for non-professionals and across disciplines.	1

Participants were asked to be as specific and practical as possible. For example, if participants recommended increased research in a particular area, they also needed to provide specific ideas that could be included in a Request for Applications and recommendations of who should conduct and fund the research (i.e., government, academia or other organizations).

#### Privacy and confidentiality (54 votes)

##### Issues

In some circumstances, such as in sparsely populated areas, the geographic location of a patient's residence in a health record can become a personal identifier. The Health Insurance Portability and Accountability Act (HIPAA) only permits the release of data with geographic identifiers smaller than the state level if "a person with appropriate knowledge of and experience with generally accepted statistical and scientific principles and methods for rendering information not individually identifiable: applying such principles and methods, determines that the risk is very small that the information could be used, along or in combination with other reasonably available information, by an anticipated recipient to identify an individual who is a subject of the information...". [26], Section 164.514 (b) (1). Because of the vague and subjective nature of this provision, many agencies take the safe approach of simply not releasing any data with geographic identifiers. Also, many states have their own privacy requirements that further constrain the release of health data.

Yet it is in the interest of overall public health activities that health data with geographic identifiers be readily available to the research community, within the limits of HIPAA regulations. For cancer control activities in particular, it is important to be able to compare cancer rates, risk behaviors, screening patterns, diagnosis stage, and treatment methods across geographical and political boundaries and at as fine a spatial scale as possible. The challenge is to find a way to make the data as widely available as possible while still protecting patient confidentiality.

##### Recommendations

1. Develop a compilation of resources that help maximize the availability of health data with geographic information. This compilation is envisioned as a web-based collection of methods, tools, sample policies, data usage agreements, and evaluation results. It should provide a guide to the "generally accepted statistical and scientific principles and methods for rendering information not individually identifiable" as specified in the HIPAA regulations. Because these principles and methods will evolve over time, resources must be allocated for ongoing maintenance of the content. Methods and tools need to be able to be tailored to the needs of users in different locations. It should include descriptions of experiences, both successful and unsuccessful. It is important to solicit feedback from the potential user community so that the collection can be continually refined and improved as it develops.

2. Support research to evaluate methods and resource requirements for the overall management of health information in a way that both protects confidentiality and maximizes availability. The research should include the evaluation of administrative costs, information technology (IT) infrastructure costs, and personnel costs. Techniques evaluated should include external release of de-identified data and internal geographic analysis of the data through software agents capable of analyzing the original data within the data repository and returning results that do not include any identifiable information[27]. Results should include an evaluation of the costs to implement various protection methods and the benefits of making the data available. Initial funding by government or other funding organizations should be directed at a specific cancer control application area.

#### Space/time problems (43 votes)

##### Issues

Current data about the spatial location of cancer patients are limited to, for the most part, the residential address at the time of diagnosis. Because most cancers have a long latency period, a history of spatial locations for some time leading up to diagnosis is needed to assess possible spatially-dependent risk factors. In addition to potential environmental exposures, these factors include socioeconomic effects, cultural differences, and access to care issues. The types of additional space-time data needed include residential history, daytime locations (work or school), and seasonal migration (winter and summer homes).

Tools and methods exist for spatial analysis and for temporal analysis but few tools and methods can be applied to both space and time together. There is a need to develop the underlying statistical theory as well as specific tools for space-time data analysis and visualization. In addition, a data representation problem needs to be solved: how to store and retrieve integrated space-time data consisting of multiple sets of data from fundamentally different space-time frames. Event-based health information, continuous sample-based environmental data, point-based health service provider locations, and area-based data on neighborhoods often need to be integrated. For example, health events (such as breast cancer occurrence) at various places and points in time might need to be integrated with soil- and air-quality samples taken from various monitoring sites in the area. For each health event, relevant environmental samples over time must be identified and a space-time interpolation from the multiple sample sites must be performed in order to estimate the environmental quality in each location at an appropriate time, considering the expected lag between exposure to the risk factor and diagnosis of the cancer. In addition, we might need to include the locations of nearby health clinics and their hours of operation, and data on neighborhood characteristics, such as income and education levels, from the decennial census. Research is needed to determine how best to store, retrieve, and process data with such widely disparate spatial and temporal frames.

##### Recommendations

1. Support an integrative program of research on geography, GIScience, spatial statistics, geo-visualization, and computer science to enable the analysis of the space-time components of cancer control. Initial program goals might include:

• Build a prototype visualization tool that would link and display cancer data with environmental data in space and time.

• Develop a space-time data model that accounts for different data types (e.g., health events and environmental time samples).

2. Support a study that investigates the feasibility of linking cancer case records with other existing data to obtain elements of a residential history and other space-time location information. Possible other data sources include: tax records, property records, Medicare records, driver's license records, school attendance records, and immigration records.

3. Fund a pilot study that collects residential history of cancer survivors. Cancer registries may have some useful residential history data available today in their tracking of cancer survivors for follow-up. Use these data to determine the operational feasibility of collecting such a history and the utility of the data for cancer control activities.

4. Host a follow-up workshop that continues the discussion of the problem of space-time data and tools for cancer control research. Given the limited time available at this workshop, participants felt that a continued discussion would generate a more complete set of solutions and next steps.

#### One stop portal (31 votes)

##### Issues

The proliferation of data and tools accessible through the Internet creates a problem for the cancer control researchers in identifying what is available and most appropriate for their research. Some data collection and geography data portals have been developed, but not all cancer control users are aware of these resources, and in some cases the tools to access the data are not easy to understand or use.

##### Recommendations

1. Form a diversified committee comprised of representatives from state, federal, and independent agencies, plus academia, user communities and other non-governmental organizations to develop a searchable and usable one-stop portal for data, boundaries, references, and tools. The committee should:

• Collaborate among data holders and GIS developers to create metadata that are standardized, understandable, and usable by multiple stakeholders.

• Identify the status of existing standards and encourage standardization of metadata and adherence to standards by data providers and GIS developers.

• List existing cancer-related portals (e.g., Geospatial One-Stop[28]) and conduct a literature review, if appropriate, to identify any additional available cancer portals. Develop an inventory/catalog of the portals, which includes metadata, related datasets, related links and supportive tools. This catalog should be "user-centric" and designed to be intuitive so that users can easily search, browse, and retrieve information.

• Create, market and promote the one-stop portal to ensure cancer control researchers are fully aware of its availability and usability; also, provide user-friendly tutorials to promote quick access and use of the portal.

2. Several participants questioned the necessity of forming such a committee, pointing to Geospatial One Stop[28] as a portal designed to meet the stated objectives. However, others argued that the usability of the existing portals could be improved and that none of them provided a comprehensive selection of data, boundaries and contributed software tools. This discussion generated a secondary suggestion that a focus group and/or usability study be conducted to evaluate the existing portals in order to provide specific suggestions for their improvement.

#### GIS centers of excellence (19 votes)

##### Issues

Workshop participants also focused on applied or translational GIS applications to promote cancer control and prevention. These types of projects must include an interdisciplinary team of scientists, including health policy experts, environmental scientists, geographers, in addition to statisticians and GIS scientists.

##### Recommendations

1. Fund a GIS Center for Excellence that includes statisticians, geographers, environmental scientists, public health scientists, health policy experts, and others. The mission of the center would be to initiate projects that focus on applied or translational GIS applications in cancer control and prevention. At least one project of the center would be a community-based participatory project. The center would develop partnerships with public health and community-based organizations and could provide an environment to foster collaboration between domain experts and GIS researchers who have methodologies that could be applied to cancer control problems. A center would have several elements (or cores):

• A research program with at least two investigator-initiated research projects (e.g., NIH R01-funded projects).

• Community outreach that would involve community-based organizations and public health practitioners, cancer registries, and other public health entities.

• A pilot program consisting of two or more investigator-initiated small research projects (e.g., NIH R03-funded projects). This program would be used to further identify important avenues of harnessing GIS projects and expertise toward the promotion of cancer control and prevention.

• Training of post-doctoral scientists, professionals, and undergraduates.

• A GIS technology program, which would further the theoretical and applied capability of this technology to cancer control and prevention by:

◦ developing geospatial tools for etiologic research;

◦ creating robust methods for measuring and mapping cancer health disparities; and

◦ evaluating cancer control interventions using GIS applications.

2. Create an intramural research center at NCI, much like a Center of Excellence. The mission of the intramural center would be to focus on theory-based GIS methods in cancer control and prevention. This intramural center, staffed by NCI researchers, would serve as a Federal partner in the proposed Centers of Excellence program (in 1, above), and would serve as a GIS resource for other units in NCI. The intramural center would conduct focused research in:

• Spatial analysis tools;

• Exposure assessment tools;

• Social-behavioral-geographic factors; and

• Data integration across the above three elements.

#### Strategies and tools for evaluating, handling, and communicating uncertainty (18 votes)

##### Issues

Standard statistical methods measure the uncertainty of a statistical estimate or test result through standard errors, confidence intervals, or p values. For example, the uncertainty of calculated cancer rates depends strongly on the underlying population sizes of the geographic areas, with rates for small areas appearing quite unstable over time. However, other sources of uncertainty are typically unmeasured or ignored. For example, the statistical measures noted above ignore the uncertainty in the choice of method, such as the choice of underlying statistical regression model. In addition and perhaps more importantly, the quality of the original cancer and other data can be uncertain. This uncertainty can be due to variability of quality of the data collection methods across registries or to errors in the assignment of case addresses to geographic location by geocoding. Other important sources of uncertainty arise when registry staff or researchers attempt to communicate technical information to policymakers or the public. Presentation of this information can be unclear or misinterpreted by the audience. More work is needed to identify the many sources of uncertainty in cancer data, incorporate this knowledge into any statistical methods, and communicate results clearly to the intended audience.

##### Recommendations

1. Increase resources to improve the quality of collected data, including:

• Registry data, such as through improvements in geocoding methods;

• Small-area demographic data and intercensal estimates, e.g., by the Bureau of the Census;

• Cancer risk factor exposure data, particularly historic data to account for the long latency of cancer development; and

• Residential histories of cancer cases to address uncertainty in exposure assessment due to population migration.

2. Support for further development, evaluation, and dissemination of robust statistical methods for handling uncertainty. These methods include:

• Improved methods for incorporating uncertainty from various sources into confidence intervals and standard errors of rates, and methods to stabilize these rates;

• Cluster identification methods that define cluster borders and underlying populations within them; and

• Methods for quantifying the uncertainty in maps presented at different levels of aggregation (related to the Modifiable Areal Unit Problem in geography and the ecologic fallacy in epidemiology).

3. Support research on sound methods for portraying and communicating uncertainty to a variety of audiences, including policymakers and communities. For example, we need to learn how to communicate to a non-technical audience that a statistically significant apparent cluster of cancer cases may not be meaningful and, conversely, that a cluster that is not significant may in fact be real. Specific recommendations include:

• Conduct research on cognitive perception of visual displays of uncertainty.

• Develop an Internet resource that accumulates best practices for handling and visualizing uncertainty.

• Hold workshops or conferences on the science of uncertainty and perception of uncertainty.

#### Methods to use census data more effectively in combination with health data (15 votes)

##### Issues

The Bureau of the Census is an important source of location-specific information for the cancer control community. Researchers frequently use data from the decennial demographic survey as well as from other surveys. In addition, the Census Bureau is a source of information on U.S. geography, particularly through the Topologically Integrated Geographic Encoding and Referencing system (TIGER) line file database. The complexity and scope of these products often make it difficult for users to locate the information required. For example, although the Bureau's newer data dissemination web sites are easy to use, it is often difficult to obtain the more complex, stratified datasets required for cancer research.

##### Recommendations

Communication between the Census Bureau and NCI and NLM needs to be improved. NCI and NLM should make a greater effort to help Bureau staff understand the geospatial data requirements of the cancer control community. In turn, the Census Bureau should take advantage of opportunities to demonstrate its current capabilities. Regularly scheduled meetings will increase understanding of all stakeholders' needs, which will lead to the development of Census Bureau activities, programs, and products that will meet the needs of the cancer control community.

#### Community-based, participatory research (14 votes)

##### Issues

Community-based participatory research (CBPR) is "research that is conducted as an equal partnership between traditionally trained "experts" and community members that are unified by a particular concern" [29] with the community participating fully in all aspects of the research. Risk assessment or community intervention studies are good examples of projects that should use a CBPR approach. As attention to this type of research grows, investigators increasingly need to develop and share strategies to ensure the successful participation of communities in their studies. For example, several town hall meetings were held on Long Island, NY, during the design and implementation of the Long Island GIS [6]. An important lesson learned from that project was to involve the community early and substantially in the study development phase and to keep residents informed throughout the study's implementation. Workshop participants used this lesson learned to formulate their recommendations in this area.

##### Recommendations

1. CBPR should be a mandated approach and funded as part of a multidisciplinary Center of Excellence that features:

• A senior investigator who has an active role in the application of CBPR within the Center;

• A community outreach and translation core;

• Health communication expertise;

• Full representation for each project from the community; community members must be sought out and engaged;

• Avenues to educate, train, and build trust within the community, including peer-to-peer mentoring, town halls, public meetings, and other forums; these forums should be tailored to the community and the intended audience.

2. Drawing on established methods for CBPR in the published literature, researchers should openly discuss proposed projects with the community in which the research will be conducted. These discussions should:

• Determine the methodologies that will be used in the projects;

• Educate the community about the benefits and limitations of GIS and its use as a research tool;

• Define areas to be covered by the research (e.g., Are the study boundaries recognized by geographers, census, community members, other advocates, or a combination? Do the various boundaries affect the study results themselves and/or the interpretation of the results by the community?);

• Determine data ownership (i.e., Do the data belong to the community, the researchers, or both?); establish rules for data use and sharing, keeping in mind that many of these projects may be funded with public money; all parties should agree to written data use rules;

• Establish methods to measure the outcomes of the project (e.g., did the project and the methodology used result in an increase in cancer screening or a reduction in risk exposure?);

• Determine through tests the elements of GIS that contribute to community change; and

• Engage community participation in research communication, systematic measurement of results, and communication and reporting of results.

#### Research and development of tools and other resources for geographically-based cancer research (9 votes)

##### Issues

In order for science to advance rapidly, it is essential to provide support for "high-risk, high-gain" research activities. Although some NCI funding mechanisms do exist for high-risk research activities (e.g., small exploratory research grants), they do not target geographically-based cancer research in particular.

##### Recommendation

1. Establish an appropriate funding mechanism that specifically targets geographically-based research questions and application and development of emerging technologies. Examples of such research questions include:

• How might data from sensor systems, such as the Earth Observing Systems, be used to better quantify space-time variability in environmental factors that play a role in carcinogenesis (e.g., air-borne particulates and lung cancer)?

• How can location-based monitors and individual sensor technologies (e.g., building sensors, micro-movement sensors) be used to enhance understanding of relationships between individual activity patterns and/or environmental exposure levels and cancer risks?

• What strategies and methods can be employed to protect confidentiality when location-based technologies are used in research?

• Can currently distributed information resources be integrated and interpolated to develop exposure risk maps and maps of the associated uncertainty? If so, how accurate are these estimates and what is their uncertainty?

#### Additional issues

Twenty-five additional issues were raised on the first day of the workshop, but did not garner enough votes for further discussion on the second day. These are shown in Table 1 (Issues #9-33). Many of these issues concerned training of cancer control staff, policy makers and others who need to process or understand geographic data, methods, and tools. Suggestions for accomplishing this objective included developing mentorship and training programs, developing and disseminating best practices for geographically-related analysis, and creating an information exchange forum on needs and solutions for users and providers of geographic data and related analytic methods.

The multidisciplinary nature of GIS led participants to call for more interdisciplinary collaboration to develop methods for working effectively with geospatial information (images as well as tabular data) to understand community patterns of cancer and to communicate information to non-professional audiences. One specific recommendation was to initiate a collaboration between the U.S. Postal Service (USPS) and other federal agencies to facilitate the use of USPS address data for improved geocoding. For example, USPS information about new streets and changing ZIP code boundaries over time could improve boundary files that are created and disseminated by other agencies and private vendors.

Several additional issues raised were related to data quality assessment and improvement. For example, the match rate and accuracy of geocoding methods need to be better understood and improved, and data compatibility across data providers could be improved. Other suggestions included for the development of GIS methods that could be used to evaluate the effectiveness of programs designed to reduce the cancer burden and the creation of user-friendly GIS tools for users of all technical levels.

### Conclusions and future directions

The enthusiastic efforts of workshop participants to provide a long list of challenges that need to be met in order to move cancer control forward is a testament to the timeliness of this workshop. The health community in general and the cancer control community in particular have only recently embraced the use of GIS methods. We are indeed at a key point in which the issues identified here need to be addressed before GIS methods can be fully used to lessen the cancer burden.

Recognizing that most of these topics are relevant across many domains, not limited to cancer control, most efficient progress on several of the issues raised can be achieved by collaboration and consensus development across federal agencies and among government, academia, cancer registries and other interested groups. For example, the call for methods to balance the need for privacy with the need for small-area data for analysis is not limited to health data. Perhaps this is one area in which a report by the National Academy of Sciences (NAS) would be warranted. Other collaborations, such as between the Bureau of the Census and its data user groups, can be more easily initiated by a new inter-agency committee or a periodic conference. Toward this end, the Census Bureau has begun a Federal Agency Information Program to help staff at other federal agencies understand and use the American Community Survey, which will replace the long form in future censuses.

In this era of limited resources for new research initiatives, follow-up workshops could be held to further define needed research on spatio-temporal data and methods and on sources of uncertainty and methods to address them. Some pilot studies or research limited in scope in these and other related areas could be carried out through small contracts, collaborative working groups, interagency agreements, and in-house research. For example, NCI and its partners could utilize existing infrastructure and contracts to conduct formative evaluations and usability testing to identify ways to improve geospatial data portals and to encourage their more widespread use by the cancer control community.

Small steps toward the more easily attainable goals have already been made since the workshop last year. For example, as a result of contacts made at this workshop, NCI and NLM are continuing their interagency discussions. NLM staff has initiated contacts with the University Consortium for GIS (UCGIS) to define bibliographic needs of this community that could be met by NLM either by indexing a greater number of GIS-related journals or by hosting a GIS-specific bibliographic database. NCI and NLM staff recently have made presentations to the Geographical Sciences and Mapping Science committees of the NAS describing GIS activities at NIH and calling for their help in convening a workshop to develop recommendations in areas of broad interest, such as privacy and confidentiality of health data, spatio-temporal data and measures of uncertainty. NCI staff also participated in a recent workshop on spatio-temporal data issues organized by the UCGIS; although focused on development of a research agenda for the U.S. intelligence community, the cross-disciplinary group of participants addressed many of the same problems identified by our cancer control experts.

The workshop accomplished a major objective by bringing many disparate groups together to identify key GIS issues and potential solutions. Following the lessons learned by SAMHSA and PSU, presented by Mr. Reynolds and Dr. Lengerich, respectively, workshop participants were encouraged to work collaboratively toward common goals that were identified; in fact, the workshop itself fostered multidisciplinary collaborative networking during the meetings. The recommendations that emerged from this meeting will be an important guiding force in helping advance a GIS and cancer control research agenda and ultimately will help reduce the future cancer burden. As these topics are applicable beyond the U.S., the results of this workshop can serve as a valuable resource for setting research agendas around the world.

## Authors' contributions

All authors participated in the planning and/or conduct of the workshop and helped to draft this manuscript. All authors read and approved the final manuscript.

## Disclaimer

The use of trade names and commercial sources is for identification only and does not imply endorsement of these products.
